# A Novel Method to Apply Osteogenic Potential of Adipose Derived Stem Cells in Orthopaedic Surgery

**DOI:** 10.1371/journal.pone.0088874

**Published:** 2014-02-19

**Authors:** Xiang Fang, Hideki Murakami, Satoru Demura, Katsuhiro Hayashi, Hidenori Matsubara, Satoshi Kato, Katsuhito Yoshioka, Kei Inoue, Takashi Ota, Kazuya Shinmura, Hiroyuki Tsuchiya

**Affiliations:** Department of Orthopaedic Surgery, Kanazawa University, Kanazawa, Japan; University of Pittsburgh, United States of America

## Abstract

**Background:**

A number of publications have reported that adipose derived stem cells (ADSCs) have the capacity to be induced to differentiate into osteoblasts both in vitro and in vivo. However, it has been difficult to use separate ADSCs for cortical bone regeneration and bone reconstruction so far. Inspired by the research around stromal stem cells and cell sheets, we developed a new method to fabricate ADSCs sheets to accelerate and enhance the bone regeneration and bone reconstruction.

**Purpose:**

To fabricate ADSCs sheets and evaluate their capacity to be induced to differentiate to osteoblasts in vitro.

**Methods:**

Human adipose derived stem cells (hADSCs) were employed in this research. The fabricating medium containing 50 µM ascorbate-2-phosphate was used to enhance the secretion of collagen protein by the ADSCs and thus to make the cell sheets of ADSCs. As the separate ADSCs were divided into osteo-induction group and control group, the ADSCs sheets were also divided into two groups depending on induction by osteogenesis medium or no induction. The osteogenic capacity of each group was evaluated by ALP staining, Alizarin Red staining and ALP activity.

**Results:**

The ADSCs sheets were fabricated after one-week culture in the fabricating medium. The ALP staining of ADSCs sheets showed positive results after 5 days osteo-induction and the Alizarin Red staining of ADSCs sheets showed positive results after 1 week osteo-induction. The ALP activity showed significant differences between these four groups. The ALP activity of ADSCs sheets groups showed higher value than that of separate ADSCs.

**Conclusion:**

The experiments demonstrated that ADSCs sheets have better capacity than separate ADSCs to be induced to differentiate into osteoblasts. This indicates that it is possible to use the ADSCs sheets as a source of mesenchymal stem cells for bone regeneration and bone reconstruction.

## Introduction

The differentiation of stem cells into the osteogenic lineage for subsequent use in osteoregeneration is a major goal in regenerative medicine. Three key factors: cells, regulatory factors and scaffolds have established their positions in this regenerative medicine field [1]. Traditionally, bone marrow stromal cells (BMSCs) have been the focus for osteoregeneration [2], and the embryonic stem cells (ES cells) are gradually gaining attention because they are also a source of regenesis stem cells [3]. Induced pluriopotent stem cells (iPSCs) also attracts attention in the regenerative medicine, and it is considered one of the promising sources of regenesis stem cells [4]. Researchers have investigated the applications of human adipose derived stem cells (ADSCs), which are recognized as one of mesenchymal stem cells, in the osteo-regenerative medicine [5].

There are several options to utilize ADSCs for repair and reconstruction of injured tissues and organs. Direct injection of ADSCs into the sites of repair is a classic method of ADSCs application [6]–[9]. The use of scaffolds composed of natural biodegradable matrices represents an attractive strategy to circumvent the lack of cell engraftment which is a major limitation of stem cell therapy in cardiovascular diseases [10]. It has been widely reported that the use of ADSCs sheets for tissue regenesis and has found successes in cardiovascular and plastic surgery fields [11]–[20].

The currently available ADSCs sheets could be generally divided into two types, one that is supported by a specific carrier such as the products of CellSeed Inc. [18]–[22], and one that makes the carrier of collagen protein in the laboratory to be transplanted together with the cell sheets to the transplant sites [10] [23]. ADSCs sheets can be transplanted with or without carrier. To our knowledge there is no attempt to use ADSCs sheets in orthopaedic surgery. Recently, there were some satisfactory reports of injection of ADSCs into the necrotic femoral head [7] [24]. It reported that the patients who were injected with ADSCs showed probable bone formation in femoral head necrosis by MRI examination. However, it is still difficult to fix the ADSCs to the cortical bone.

Our orthopaedic laboratory has developed liquid nitrogen therapy that preserves the bone matrix after we froze the bulk of bone in liquid nitrogen and then returned and fixed it to the original location [25]–[27]. The existing application of ADSCs in the orthopedic field is to inject the mixture of ADSCs and other materials such as normal saline, fibrin gel and collagen gel into the spongy bone [7] [24]. It is almost impossible to inject the mixture into the cortical bone.

Ascorbate-2-phosphate has been reported to increase the collagen protein-secretion of the mesenchymal cells without any other effect to the cells [28]. Collagen protein has been considered as a good natural carrier of cell sheet in osteogenic lineage [29].

Integrating the above information, we sought to combine the liquid nitrogen frozen bone autografts scaffolds and ADSCs sheets together to accelerate and enhance the bone regeneration and bone reconstruction. Compared with other existing ADSCs sheets, our new method does not employ other materials besides ascorbate-2-phosphate, thus reduce the cost and increase the grafts histocompatibility. It is the first step of this research that the in vitro experiments of inducing the ADSCs sheets to differentiate into osteoblasts and to confirm the cells of the ADSCs sheets keep the capacity of being induced to differentiate into osteoblasts. A series of subsequent studies have been designed including measuring the mechanical strength of the ADSCs sheets, testing the in vivo safety of the ADSCs sheets and reducing the xenogeneic factors in the medium. The outcomes of this in vitro study revealed that the ADSCs sheets presented higher osteo-differentiation capacity than separate ADSCs. These pretreated mesenchymal stem cells could be a better effective autograft for bone regeneration.

## Materials and Methods

### 2.1 Materials

Human Adipose Derived Stem Cells (ADSCs) from Life Technologies (Japan) were selected for the experimental material. It has been shown that the ADSCs express a flow cytometry cell surface protein profile as follows: positive CD29, CD44, CD73, CD90, CD105, CD166; negative CD14, CD31, CD45 and Lin1. The vial of ADSCs was delivered to the laboratory in a dry ice box and it was immediately preserved in the liquid nitrogen tank. A series of experiments were done to confirm that these cells retain the capacity to be induced to differentiate to osteoblasts.

### 2.2 Methods

#### 2.2.1. Preparing hADSCs

The hADSCs were suspended in DMEM (Wako Pure Chemical Industries, Ltd.) supplemented with 10% fetal bovine serum (FBS) (NICHIREI BIOSCIENCES, INC.) and 1% Penicillin-Streptomycin Solution (P/S) (Wako Pure Chemical Industries, Ltd.), and incubated at 37°C for 1day.

#### 2.2.2. Culturing the hADSCs

The change of the activated hADSCs’ shapes from round to spindle shape could be observed by the optical microscope after 1 day of incubation. The changes of their shapes were due to the secretion of collagen fibrous protein from the woke-up cells and the cells which secreted the collagen fibrous protein were able to attach to the bottom of the petri dishes. The petri dishes were gently rinsed 3 times by prewarmed PBS (Wako Pure Chemical Industries, Ltd.) to remove those unattached cells. The remaining hADSCs were cultured in DMEM (Wako Pure Chemical Industries, Ltd.) supplemented with 10% fetal bovine serum (FBS) (NICHIREI BIOSCIENCES, INC.) and 1% Penicillin-Streptomycin Solution (P/S) (Wako Pure Chemical Industries, Ltd.) [30] [31], and been subcultured until they grew to covering 90% of the area of the petri dishes. The passage 3 hADSCs were prepared for the subsequent experiment.

#### 2.2.3. Inducing the hADSCs to differentiate to osteoblasts

The hADSCs at passage 3 were analyzed for their capacity to differentiate towards the osteogenic pathway. To induce differentiation, the hADSCs were cultured with specific induction media. The osteogenic medium (OM) was composed of α-MEM (Wako Pure Chemical Industries, Ltd.) containing 10% FBS, 0.1 µM dexamethasone, 50 µM ascorbate-2-phosphate, 10 mM β-glycerophosphate, 1% Penicillin-Streptomycin Solution (P/S) [5] [32]–[35]. α-MEM solution containing 10% FBS, 1% Penicillin-Streptomycin Solution (P/S) was used as the control medium for each condition.

#### 2.2.4. Staining the induced cells with alkaline phosphatase staining kit and alizarin red staining kit

Alkaline phosphatase histochemistry was performed at 1 d, 3 d, 5 d, 7 d, 10 d, 2 w, and 3 w during the induction culture [31] [36] [37]. When the ALP staining was done, the medium was removed, and the cell layers were rinsed with PBS 3 times and fixed in 4% Paraformaldehyde Phosphate Buffer (Wako Pure Chemical Industries, Ltd.) for 5 minutes at room temperature. After 5 minutes at room temperature, the cell layers were washed with deionized water. Then, the fixed cells were incubated with 1-Step NBT/BCIP plus Suppressor Solution (Thermo Fisher Scientific INC.). After 30 minutes incubation at 37°C, the cell layers were washed with deionized water and observed both grossly and with the light microscope.

Before Alizarin Red staining, the cell layers were rinsed with PBS 3 times and fixed in 4% Paraformaldehyde Phosphate Buffer (Wako Pure Chemical Industries, Ltd.) for 10 minutes at room temperature. Then the cells were washed with deionized water for 3 times. Then the Alizarin red staining was done following the standard protocol as described in the Osteogenesis Assay Kit (ECM815, Millipore) [38].

#### 2.2.5. Fabricating the hADSCs sheets

The passage 3 hADSCs were seeded in the 10 cm petri dishes by 1×10^6^ cells/dish and were cultured in DMEM (Wako Pure Chemical Industries, Ltd.) supplemented with 10% fetal bovine serum (FBS) (NICHIREI BIOSCIENCES, INC.) and 1% Penicillin-Streptomycin Solution (P/S) (Wako Pure Chemical Industries, Ltd.) until over confluent for fabricating the hADSCs sheets. The fabricating medium contained 10% FBS, 50 µM ascorbate-2-phosphate, 1% Penicillin-Streptomycin Solution (P/S). The mediums were changed every 3 days in 1 week. This is the key step that we developed to make the new ADSCs sheets. Samples were randomly picked and checked to make sure if the ADSCs sheets were fabricated.

#### 2.2.6. Inducing the fabricated cell sheets to differentiate to osteoblasts

To induce osteo-differentiation, the hADSCs sheets were cultured with specific induction media. The osteogenic medium (OM) was composed of α-MEM (Wako Pure Chemical Industries, Ltd.) containing 10% FBS, 0.1 µM dexamethasone, 50 µM ascorbate-2-phosphate, 10 mM β-glycerophosphate, 1% Penicillin-Streptomycin Solution (P/S). α-MEM solution containing 10% FBS, 1% Penicillin-Streptomycin Solution (P/S) was used as the control medium for each condition.

#### 2.2.7. Staining the ADSCs sheets with ALP staining kit and alizarin red staining kit

Alkaline phosphatase histochemistry was performed at 1 d, 3 d, 5 d, 7 d, 10 d, 2 w, and 3 w during the induction culture period. When the ALP staining was done, the medium was removed, and the cell layers were rinsed with PBS 3 times and fixed in 4% Paraformaldehyde Phosphate Buffer (Wako Pure Chemical Industries, Ltd.) for 5 minutes at room temperature. After 5 minutes at room temperature, the cell layers were washed with deionized water. Then, the fixed cells were incubated with 1-Step NBT/BCIP plus Suppressor Solution (Thermo Fisher Scientific INC.). After 30 minutes incubation at 37°C, the cell layers were washed with deionized water and observed both grossly and with the light microscope.

Before being Alizarin Red stained, the ADSCs cell layers were rinsed with PBS 3 times and fixed in 4% Paraformaldehyde Phosphate Buffer (Wako Pure Chemical Industries, Ltd.) for 10 minutes at room temperature. Then the cells were washed with deionized water for 3 times. Then the Alizarin red staining was done following the standard protocol as described in the Osteogenesis Assay Kit (ECM815, Millipore).

#### 2.2.8. Quantification of ALP activity

ALP activity of ADSCs groups and ADSCs sheet groups were determined using TRACP & ALP assay kit (MK301, TaKaRa Bio, Japan) according to the manufacturer’s instruction. The check points were set at Day 1, 3, 7, 10, 14 and 21.

#### 2.2.9. Statistical analysis of ALP activity

Student’s t-tests were used to statistically assess the significance of differences of ALP activity between the four groups at the same time. Comparisons with p-values lower than 0.05 were considered significant.

## Results

**Figure 1:**
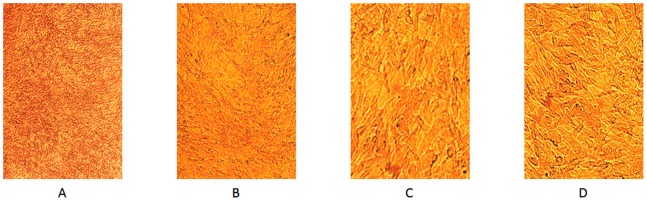
4×microscopic view(A) shows the over fluent state of ADSCs sheets. 10×microscopic view(B) shows there is no space between the ADSCs of the sheets. 20×microscopic view(C) shows the shape of the ADSCs of the sheets are similar to separate ADSCs(D).

The ADSCs and ADSCs sheets were evaluated by optical microscope. And the changes of the cell shapes were observed during the experiments. Actually, the cell number were unchanged after achieving the over confluent status. The ADSCs sheets could be observed clearly under optical microscopes (Fig. 1).

**Figure 2:**
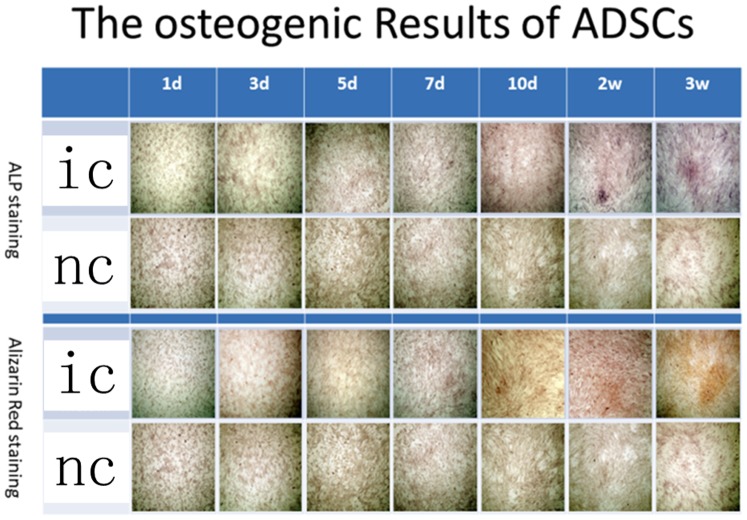
Both ALP staining and Alizarin Red staining proved that ADSCs can be induced to differentiate to osteoblasts. ic: ADSCs in osteo-induction media, nc: ADSCs in common media.

**Figure 3:**
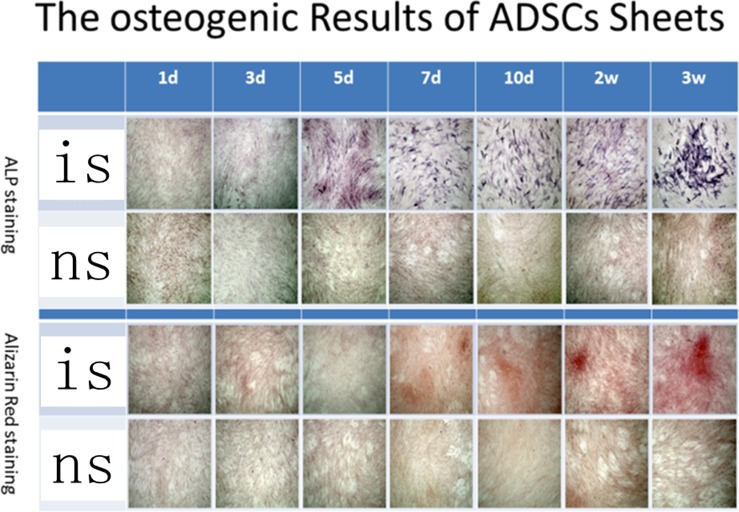
Both ALP staining and Alizarin Red staining proved that ADSCs sheets can be induced to differentiate to osteoblasts. is: ADSCs sheets group in osteo-induction media; ns: ADSCs sheets group in common media.

The differences of the results of ALP staining between the hADSCs control groups and induced hADSCs groups were shown at 2 weeks since the induction started and the differences of the results of Alizarin Red staining between these two groups were showed at week 3 (Fig. 2). In contrast, the differences of the two hADSCs sheets groups were showed at day 5 of induction by ALP staining and at day 7 by Alizarin Red staining (Fig. 3).

The concentration of both ALP staining and Alizarin Red staining were getting deeper over time. At each corresponding check point of staining, the concentration of ALP staining and Alizarin Red staining of induced hADSCs sheets groups were deeper than that of separate hADSCs (Fig. 2, 3).

**Figure 4:**
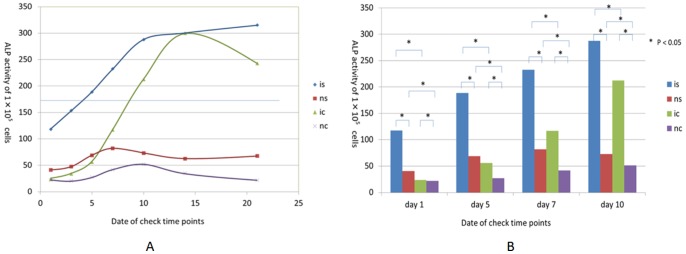
ALP activity curves of separate ADSCs and ADSCs sheets under osteo-induction condition and compared with control groups. (A) ALP activity of separate ADSCs and ADSCs sheets under osteo-induction condition are compared with control groups on day 1, 3, 5, 7. (B) is: ADSCs sheets group in osteo-induction media; ns: ADSCs sheets group in common media; ic: ADSCs in osteo-induction media; and nc: ADSCs in common media.

The mean values of ALP activity of separate ADSCs shows that the ALP activity of ADSCs grew rapidly since day 5 of osteo-induction. Coinciding with the User Manual of ADSCs from Life Company, this increasing achieved the extreme value on day 14, which is consistent with other published studies [39]. The ALP activity of the separate ADSCs control group kept a low value range during the whole experiment period. Both the ADSCs sheet osteo-induction group and the ADSCs sheet control group shows higher ALP activity at day 1 compared with the separate ADSCs groups (P<0.05). Especially the ADSCs sheet osteo-induction group showed a significantly higher value than separate ADSCs groups (P<0.05). The linear increase of the ALP activity of ADSCs sheet osteo-induction group started from day 1 and got close to the extreme at day 10. However, the ALP activity of the ADSCs sheet osteo-induction group was still increasing at day 14 and day 21. Although the ALP activity of the ADSC sheet control group kept a lower value range during the whole experiment period compared with the ADSCs sheet osteo-induction group, the values are still larger than that of the separate ADSCs control groups (P<0.05) (Fig. 4).

## Discussion

Our study demonstrated that the novel ADSCs sheets could be induced to differentiate into osteoblasts as well as separate ADSCs. This new method to apply ADSCs in regenerative medicine of orthopaedic surgery provides a new mode combining regenerative cells and fixation, moreover, this method up-regulates the osteogenic capacity of ADSCs.

With the osteogenic medium, the changes of the cell shapes could be observed by optical microscope during the period of induction. At the check points of day 15 and day 21, the ALP staining and Alizarin Red staining showed the high activity of ALP and calcium precipitation. These indicated that the osteogenic differentiation had already been inducted. It is identical to the results of published papers (15 to 18 days). The outcomes had been confirmed by other published papers that those osteogenic medium was effective to induce the hADSCs to differentiate to osteoblasts.

According to some publications, osteoblasts could be classified as a specific kind of fibroblasts and collagen protein is part of the secretions of osteoblasts [40]. Molecular biology and genetic studies have revealed the osteogenic effects of ascorbic acid [41]–[44]. This could explain why the ADSCs sheets can keep the capacity to be induced to differentiate to osteoblasts and be induced faster to differentiate to osteoblasts than separate ADSCs. This may also explain why that the ADSCs sheets could not be digested to separate cells in 3 to 5 minutes by Trypsin like normal ADSCs but could be digested by Collagenase.

Because the ascorbate-2-phosphate in the fabricating medium enhanced the collagen secretion and the typeIcollagen could affect the osteo-differentiation of ADSCs, the ALP activity of both ADSCs sheet groups show higher values on day 1 compared with separate ADSCs groups. This sheet-fabricating step shows significant pre-warming effect when the osteo-induction started. The ALP activity of the ADSCs sheet osteo-induction group is higher than that of the separate ADSCs group on each time point and proves that the ADSCs sheets are more effective in osteogenesis compared with separate ADSCs. When considering using translating these findings to the in vivo experiments and clinical treatments, this advantage might be beneficial because the grafts of ADSCs sheets can play a role in osteogenesis sooner than the individual cells after transplantation. Moreover, the ALP activity curves (Fig. 4) shows that the ALP activity of the ADSCs sheet osteo-induction group continues to increase until the last check point of day 21; in contrast, the ALP activity shows a declining trend after day 14. This indicates that the ADSCs sheets have not achieved at their limit of osteo-differentiation, and the ADSCs sheets might have better osteogenesis capacity than separate ADSCs.

Also because of the secretion of collagen proteins, the ADSCs sheets exhibit remarkable mechanical strength that they can be directly held by ordinary experimental tweezers and ordinary surgical equipment [40] [45]. We are planning to design a series of experiments to reveal the relevant factors associated with the mechanical strength of the ADSCs sheets in the process of culturing and fabricating the ADSCs sheets. The dose of ascorbic acid in the fabricating medium has been considered as a key factor. However, experiments are needed to decide the exact appropriate dosage of ascorbic acid that could make the ADSCs sheets reach the balance point of the capacity to be induced to differentiate to osteoblasts and the mechanical strength.

This novel method to fabricate ADSCs sheets and its use provides a new consideration and prospect for bone regeneration and bone reconstruction. It breaks the limitation of the application of ADSCs in orthopedic field. It was reported that injection of the ADSCs into the osteonecrosis location of the femoral head has shown positive treatment effect. Some publications also reported that the combined use of b-TCP based bone cement scaffold and ADSCs was promising for bone defects [41] [46]. Because the ADSCs always exist as a component with a certain solution like fibrin sealant for the fixation of the ADSCs in the field of orthopedics, the concentration of ADSCs in the mixture is limited. Since now, the ADSCs are not only used as a component of a mixture but they themselves are going to be the single ingredient to attach to the scaffold to regenerate and to reconstruct the bone. The size of the ADSCs sheets can be designed and controlled in the laboratory. Moreover, through specific operations, we can remodel the ADSCs sheets to the required shape. According to the experiment planning and research progress of our laboratory, liquid nitrogen treated bone grafts [21] is going to be the scaffolds for the ADSCs sheets.

Compared with separate ADSCs, the *in vitro* induction periods of the cells of the ADSCs sheets are significantly shorter (5 days VS. 2 weeks by ALP staining and 7days VS. 3 weeks by Alizarin Red staining). And the sheet-fabricating step is considered as an effective preparation of osteo-induction when observing the ALP activity curves of the four groups. Therefore, it is reasonable to speculate that the ADSCs sheets have a higher efficiency to regenerate and reconstruct the bone *in vivo*. Moreover, the cells are not going to outflow with the degradation of fibrin sealant or with the diffusion of the solution. The local ADSCs amount could be controlled, thus the effect of ADSCs treatments could be expected.

The safety of application of the ADSCs and ADSCs sheets in orthopedic field still needs to be verified [43] [44]. We are going to use ADSCs as a source of mesenchymal stem cells to provide osteoblasts for the bone regeneration and bone reconstruction, meanwhile, the possibility of tumurogenesis affected by ADSCs must be taken into consideration. According to some publications, the secretion of VEGF and other cytokines might cause neoplasm onset. The safety of ADSCs sheets is going to be verified with further research [47]–[50].

## Conclusion

The ADSCs sheets keep the osteo-differentiation capacity as the ADSCs. Actually, the ADSCs sheets show stronger osteo-differentiation capacity than separate ADSCs. The ADSCs sheets is a new method of using ADSCs in orthopedic field which could provide a promising application together with new scaffolds such as liquid nitrogen frozen bone autografts, iodine supported titanium instruments, and bone substitutes.
